# Perceiving Animacy in Own-and Other-Species Faces

**DOI:** 10.3389/fpsyg.2019.00029

**Published:** 2019-01-23

**Authors:** Benjamin Balas, Amanda Auen

**Affiliations:** ^1^Department of Psychology, North Dakota State University, Fargo, ND, United States; ^2^Center for Visual and Cognitive Neuroscience, North Dakota State University, Fargo, ND, United States

**Keywords:** face recognition, animacy perception, perceptual learning, social vision, expertise

## Abstract

Though artificial faces of various kinds are rapidly becoming more and more life-like due to advances in graphics technology (Suwajanakorn et al., 2015; Booth et al., 2017), observers can typically distinguish real faces from artificial faces. In general, face recognition is tuned to experience such that expert-level processing is most evident for faces that we encounter frequently in our visual world, but the extent to which face animacy perception is also tuned to in-group vs. out-group categories remains an open question. In the current study, we chose to examine how the perception of animacy in human faces and dog faces was affected by face inversion and the duration of face images presented to adult observers. We hypothesized that the impact of these manipulations may differ as a function of species category, indicating that face animacy perception is tuned for in-group faces. Briefly, we found evidence of such a differential impact, suggesting either that distinct mechanisms are used to evaluate the “life” in a face for in-group and out-group faces, or that the efficiency of a common mechanism varies substantially as a function of visual expertise.

## Introduction

Face animacy, by which we refer to the appearance of a face as looking real or alive, is robustly perceived by observers (Farid and Bravo, 2011). Specifically, whether we consider artificial faces that are doll-like (Looser and Wheatley, 2010; Balas and Horski, 2012) or computer-generated (Cheetham et al., 2011; Balas, 2013; Balas and Tonsager, 2014), observers are capable of telling the difference between a real face and a synthetic one. The differences between real and artificial face appearance support basic category discrimination (Koldewyn et al., 2014; Balas et al., 2018) and also impact a number of other judgments regarding social characteristics and other more complex inferences based on physiognomy.

The animacy of a face is thus something that observers are capable of measuring, and the outcome of their measurement can influence a range of subsequent judgments about a face. Besides being another example of a categorization task we can ask observers to carry out with face images, why should we continue to examine the nature of animacy perception? What makes it theoretically important? Compared to other categories (race, gender, or species), we suggest first of all that animacy perception is unique in that categorizing a face as animate or alive further implies that an agent has a mind. That is, animate faces have thoughts, feelings, desires, and motivations, while inanimate faces do not. Faces that are more doll-like are rated as less likely to feel pain or have motives, for example (Looser and Wheatley, 2010), demonstrating that participants tend to discount the potential for artificial-looking faces to have emotions and mental states. This inference has important consequences for a number of social and perceptual processes. For example, Wykowska et al. (2014) demonstrated that attentional orienting was influenced by observers’ beliefs about the source of gaze behavior that provided an orienting cue: If the source was believed to be human, ERP responses to cue validity were enhanced relative to invalid cues, but these effects were reduced if the source was believed to be a robot. Computer-generated artificial faces that are identity-matched to real individuals, thus guaranteeing close resemblance between images, are also not socially evaluated in the same way as real faces. Perceived trustworthiness is not generally preserved across real and artificial versions of the same person (Balas and Pacella, 2017), and more broadly the position of artificial faces in a social “Face Space” defined by trustworthiness and dominance (Oosterhof and Todorov, 2008) differs from that of real faces (Balas et al., 2018). Recognizing that a face is not real thus influences a range of judgments that govern how we evaluate social agents and how we may choose to interact with them (Hortensius and Cross, 2018). Understanding how faces are perceived to be animate or inanimate is therefore also an important step toward understanding how a range of social information is subsequently processed.

Another important theoretical issue, however, has to do with how animacy perception fits into face perception models more broadly. Specifically, to what extent is animacy perception best characterized as a bottom–up or a top–down process in terms of how animacy categorization fits into other aspects of face processing? Naively, one might imagine that animacy is inferred from low-level visual features independently from other parallel processes (e.g., emotion) and thus cannot easily influence other face categorization tasks. However, bidirectional interactions between mind perception and perceptual tasks have been identified in a number of studies (see Teufel et al., 2010 for a review) and used to argue that animacy perception may result both from bottom-up interpretation of sensory stimuli and top–down contributions from neural loci responsible for Theory-of-Mind processing. Understanding the manner in which animacy categorization is carried out in perceptual tasks thus has the potential to help clarify the limitations on animacy categorization relative to low-level stimulus factors, which may help reveal the contributions that bottom-up processing can possibly make in different contexts.

Finally, understanding animacy perception is important because social interactions between an observer and an agent depend heavily on what the observer believes about the agent’s capability to think, feel, and reason. The quality of those interactions will therefore be dictated in large part by how mind perception follows from animacy perception, or vice-versa. The so-called “uncanny valley” effect is a prime example of how confusion about this relationship can lead to an unwelcome feeling of creepiness or dread based on inanimate experience coupled with the ability to have experiences (Gray and Wegner, 2012). Certain experiences can also enhance mind perception, however, further suggesting that understanding the nature of bottom-up processing of animacy in face images may be critically important to understanding the richer set of processes that lead to animacy and mind perception. For example, artificial faces rendered so that they appear to have injuries or that they are experiencing pain tends to enhance mind perception (Swiderska and Kuster, 2018), suggesting that there are multiple paths to the perception of animacy, mind, and agency. We suggest therefore that one important approach to understanding these complex interactions is to establish several basic properties of how animacy categorization unfolds in perceptual tasks. In particular, we argue that understanding the specificity of animacy categorization processes to human faces is of critical importance, considered both in terms of low-level and high-level stimulus variables.

Do judgments of face animacy largely depend on mechanisms that are specific to faces (as opposed to objects or materials) or to face categories (e.g., specific to face race or age)? There are several results that demonstrate that perceived animacy *interacts* with other face categories, which in turn suggests that face animacy mechanisms are not applied in the same manner to all faces. This in turn, has implications for how subsequent social judgments that depend on animacy are influenced by other orthogonal face categories. Perceived animacy depends on gender, for example (Balas, 2013), such that artificial faces tend to look more feminine, and feminine faces tend to look more artificial. This relationship may be the result of covariance between features that define femininity and artificial appearance, but has profound consequences for how participants evaluate male vs. female faces socially (Roylance et al., 2017). Perceived animacy also depends on race: Face images that have been morphed along an animacy continuum need a greater proportion of real face appearance to be judged as real if they are other-race faces, an effect that is observed for both adults (Hackel et al., 2014) and children (McLoughlin et al., 2017). These outcomes strongly suggest that animacy perception works somewhat differently across face categories, possibly as a function of perceptual tuning that follows from biased experience with a subset of face categories, but also potentially as a function of social connectedness relative to in-groups (Powers et al., 2014). Again, these results demonstrate that inferences regarding intentionality, agency, and the capacity to have sensory experiences (all of which appear to follow from animacy judgments) will vary across face categories.

While such results are intriguing, they don’t allow us to make particularly strong statements about the selectivity of the mechanisms involved, however, at least not in terms of well-defined cognitive or neural systems. Instead, we argue that these previous results mostly tell us that something like response criterion varies as a function of face categories (which is interesting in its own right) but don’t speak to potential differences in the underlying mechanisms that support how face animacy is measured and perceived in different kinds of faces. To do this, we need to more closely examine face animacy perception using tools that allow us to characterize the contribution of distinct visual and cognitive processes to the task of judging whether faces are alive or not.

Recently, several reports have directly addressed the issue of whether or not “expert” face processes, including configural processing, may be involved in the perception of animacy in face images. We suggest that such studies both provide useful data regarding the extent to which animacy perception depends on face-specific mechanisms and also provide a means for comparing the way animacy perception works in different categories of faces. For example, Crookes et al. (2015) examined the extent to which the other-race effect (ORE) could be measured using real and computer-generated (CG) faces, finding that there were substantial memory deficits for real faces compared to CG faces (consistent with Balas and Pacella, 2015) and also that the ORE was difficult to measure with CG faces. They considered these outcomes as evidence against CG faces tapping expert-level mechanisms for face perception. Another approach to examining the role of expert-level processing in the context of real and artificial faces is to attempt to disrupt face perception via face inversion and examine the impact of this manipulation on the processing of face animacy itself, or the processing of other aspects of appearance as a function of face animacy. Face inversion appears to have relatively modest effects on animacy categorization in some contexts (Deska et al., 2017; Balas et al., 2018), but has a measurable impact on performance in lexical decision tasks (LDT) in which faces serve as primes for human- or machine-related words (Hugenberg et al., 2016). Critically, this latter result not only demonstrated the role of upright face appearance in priming LDT performance, but further demonstrated that human/animal judgments were differentially affected by inversion. Thus, despite some evidence that chimpanzee faces are to some extent “special” to human observers (Taubert, 2009), there is at least some indications that judgments of animacy may be selective for both face orientation and face category. Taken together, all of these results suggest that artificial faces may not have the same status as real ones in terms of face-specific cognitive mechanisms and neural loci. In turn, this may mean that artificial faces are somewhat impoverished in terms of how they are represented and evaluated in the visual system. To link this to observers’ decisions about the presence of a mind within a social agent and subsequent choices regarding how to interact, these fundamental issues regarding how face animacy works at the level of visual processing may profoundly constrain the potential for artificial agents to truly function as fully social agents rather than limited proxies. The way visual processing supports and constrains animacy processing is a sort of bottleneck for how a wide range of subsequent social behaviors play out (Teufel et al., 2010), so understanding these early steps in more detail is critically important. More specifically, we suggest that these results point toward the need for more direct investigation of the nature of animacy perception in human and non-human faces, specifically with regard to the impact of stimulus-level manipulations of appearance that are known to disrupt expert-level face processing.

In the current study, we examined the selectivity of face animacy perception through the lens of the face inversion effect (Yin, 1969) and varying presentation times for face stimuli. We were specifically interested in determining how perceived animacy was affected by inversion and presentation time for (1) human faces morphed between real and doll appearance, and (2) dog faces morphed between real and doll appearance. By examining these stimulus factors, we are able to assess several fundamental questions regarding face animacy. With regard to face inversion, we chose this manipulation to closely examine how face-specific processing may contribute to animacy perception as a function of expertise with own- and other-species categories. With regard to stimulus duration, we chose to manipulate the amount of time that observers were able to see face images to see if face animacy can be judged from “thin slices” of stimulation (Ambady and Rosenthal, 1992), and if the way in which animacy is judged changes when more information is available. Finally, comparing performance with human faces to dog faces allows us to make comparisons across face categories that differ both in terms of raw experience and also in terms of the nature of social interactions between observers and agents. In prior work, we have found some evidence for independent neural mechanisms supporting the perception of animacy in human faces and dog faces (Balas and Koldewyn, 2013; Koldewyn et al., 2014), making dog faces a particularly useful point of comparison. While dogs are highly social domestic animals that have had a long history of interaction with humans (Hare and Tomasello, 2005), dog faces nonetheless are nearly always under-represented in the visual environment relative to human faces, making distinctions between dog and human face processing meaningful for studying effects of expertise (Diamond and Carey, 1986) Our key hypothesis was that animacy perception would be affected by inversion and presentation time differently as a function of species category (as evidenced by interactions of these factors with species), suggesting that distinct visual or cognitive mechanisms support animacy judgments for these face categories. That is, if face animacy is tuned to human faces, then we expected to see a differential impact of face inversion and presentation time on animacy judgments made for human faces as compared to dog faces.

## Experiment 1

In our first experiment, we investigated how presentation time and inversion affected observers’ categorization of faces according to animacy. Specifically, we examined how morphed human faces spanning a real-doll continuum were labeled as real or artificial as a function of these two stimulus parameters.

### Methods

#### Subjects

We recruited a sample of 17 observers (10 female) to take part in this experiment. We determined that this sample size would be adequate for detecting effects of face inversion and presentation time based on a power analysis that was informed by prior studies reporting medium to large effect sizes for these manipulations in other contexts. Participants were recruited from the NDSU Undergraduate Psychology Study Pool and received course credit for participating. All participants were between 18 and 26 years of age and self-reported normal or corrected-to-normal vision. Prior to the beginning of the testing session, we obtained written informed consent from all participants.

#### Stimuli

We used a set of morphed human/doll faces previously described in Koldewyn et al. (2014). Briefly, these stimuli were comprised of 256 × 256 grayscale images depicting cropped human and doll faces. Each face was displayed on a medium-gray background and all faces were female. Morphed images were created by pairing each human face with a visually similar doll face in NorrKross MorphX, and labeling matched fiducial points on both faces. The full stimulus set was comprised of 4 unique human-doll morph continua with 10 steps (11 images) spanning the real/doll endpoints, for a grand total of 44 images.

#### Procedure

Participants completed the task seated approximately 35 cm away from a 1024 × 768 LCD monitor in a behavioral testing suite with normal lighting. At this viewing distance, our face stimuli subtended approximately 4° of visual angle, though this varied somewhat across participants. Participants were asked to categorize each face as either a “real” face or a “doll” face using the ‘z’ and ‘m’ keys on the keyboard, respectively, and began the testing session when they were ready by pressing the spacebar.

During the testing session, stimuli were presented in a pseudorandomized order that was generated for each participant using the Shuffle.m functions in the Matlab Psychophysics Toolbox v3. On each trial, participants were first presented with a fixation cross for 500 ms, followed by a single face presented either upright or inverted at the center of the screen for either 160, 320, or 640 ms. We chose these values based on the range of image durations reported in previous studies of animacy perception (Looser and Wheatley, 2010; Balas and Tonsager, 2014; Balas et al., 2018) and also guided by prior results demonstrating that more complex social variables (e.g., competence) can be measured at above chance levels from face images with an image duration of 100 ms (Willis and Todorov, 2006). This face was immediately followed by a noise mask depicting fractal noise with a 1/f power spectrum, which was displayed for 500 ms before it was erased (Figure 2). Participants were asked to respond after the mask disappeared, and had unlimited time to do so. Each image was presented eight times in each condition, for a total of 2,112 trials in the entire testing session. All stimulus display and response collection routines were controlled by custom scripts written using the Psychtoolbox v3 extensions for Matlab (Brainard, 1997; Pelli, 1997; Kleiner et al., 2007). All experimental procedures, including procedures for obtaining informed consent, were approved by the NDSU IRB in accordance with the principles outlined in the Declaration of Helsinki.

### Results

**FIGURE 1:**
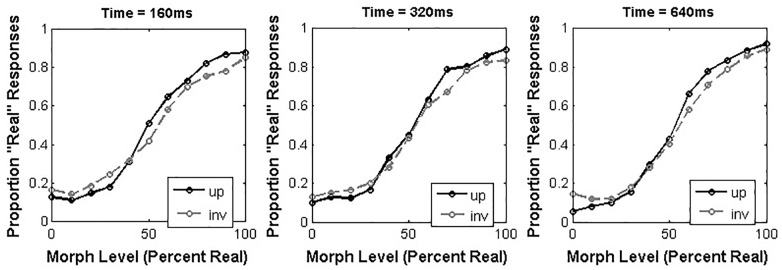
Average proportion “Real” responses to morphed human/doll face stimuli as a function of orientation and presentation time.

For each participant, we calculated the proportion of “Real” responses elicited by stimuli at each morph level as a function of both presentation time and orientation (Figure 1). Because we expected that participants’ responses across morph levels would be generally consistent with a sigmoidal shape, we used curve-fitting to describe each participant’s responses across morph levels within each combination of presentation time and orientation. Specifically, we used the Matlab ‘cftool.m’ function to obtain the best fit of each participant’s data to a cumulative normal function, which was defined in terms of free parameters governing the two horizontal asymptotes of the curve (gamma and lambda), the midpoint of the curve (alpha) and the steepness of the linear region of the curve (beta).

**FIGURE 2:**
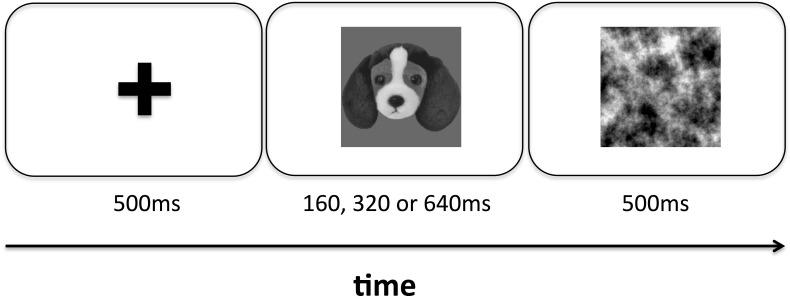
Schematic view of the time course for a single trial in both experiments. Participants viewed a fixation cross for 500 ms, followed by a single image (human faces in Experiment 1 and dog faces in Experiment 2) that was presented either upright or inverted for either 160, 320, or 640 ms. Immediately following this image, participants viewed a noise mask for 500 ms before making a response.

Thus, from each participant, we obtained four parameters (alpha, beta, gamma, and lambda) estimated from the data within each condition. The gamma and lambda parameters reflect the guessing rate and lapse rate, respectively, which we expected would be small in all conditions. We thus chose to focus our analysis on the alpha and beta parameters, which reflect the midpoint of the sigmoidal curve and the steepness of its linear region, respectively.

#### Alpha Results

We analyzed the alpha values obtained from our participants across all conditions using a 3 × 2 repeated-measures ANOVA with presentation time (160, 320, and 640 ms) and orientation (upright or inverted) as within-subjects factors. This analysis revealed a main effect of orientation [*F*(1,16) = 6.86, *p* = 0.019, $\eta_{p}^{2}$ = 0.30], such that inverted faces (*M* = 4.18, 95% CI = [-0.40,8.75]) had a larger alpha value than upright faces (*M* = 1.42, 95% CI = [-3.72,6.55]; 95% CI of the difference between means = [0.53,5.00]). Neither the main effect of presentation time [*F*(2,32) = 2.01, *p* = 0.15, $\eta_{p}^{2}$ = 0.11], nor the interaction between these factors [*F*(2,32) = 2.00, *p* = 0.15, $\eta_{p}^{2}$ = 0.11] reached significance.

We also included an exploratory analysis in which we included participant sex (male or female) as a between-subjects factor. This was motivated by our use of female human faces, which could lead to out-group effects for male participants viewing female stimuli. However, in this additional analysis neither the main effect of participant sex [*F*(1,15) = 0.33, *p* = 0.57, $\eta_{p}^{2}$ = 0.02] nor any interactions between gender and our other factors reached significance.

#### Beta Results

Like our alpha values, we also analyzed the beta values obtained from our participants using a 3 × 2 repeated-measures ANOVA with the same factor structure. This analysis revealed that neither of the main effects [orientation: *F*(1,16) = 2.04, *p* = 0.17, $\eta_{p}^{2}$ = 0.11; presentation time: *F*(2,32) = 0.88, *p* = 0.42, $\eta_{p}^{2}$ = 0.052], nor the interaction between these factors [*F*(2,32) = 1.78, *p* = 0.18, $\eta_{p}^{2}$ = 0.10] reached significance.

### Discussion

The results of Experiment 1 suggest that the orientation of morphed human/doll faces affects animacy categorization such that the point of subjective equality for inverted faces is shifted toward the “Real” end of the spectrum relative to upright faces. This is at odds with previous results (Balas et al., 2018) indicating that orientation generally does not impact psychometric curves for animacy categorization, so how do we explain the discrepancy? An important distinction between Experiment 1 and Balas et al. (2018) is the inclusion of longer presentation times, which may indicate that orientation only affects animacy categorization when images are presented for sufficiently long periods of time. We note, however, that we did not observe the critical interaction between presentation time and inversion that would make the strongest case for the dependence of an orientation effect on image duration.

Another intriguing feature of the data from Experiment 1 that differs from previous reports is the position of the PSE relative to the physical midpoint of the morph continua we created. In several previous reports, the PSE for animacy ratings has been found to be shifted rightward relative to the physical midpoint, suggesting a conservative boundary for animacy judgments. In Balas et al. (2018), we noted that under fairly rapid viewing (200 ms per image), the PSE was not significantly different from the physical midpoint, which we suggested could be the result of asking participants for a categorization judgment rather than an animacy rating. In the present study, we note that the only single cell in which we observed a significant rightward shift relative to the physical midpoint was the data from the inverted condition with a presentation time of 640 ms (95% CI = [1.47 – 11.6]). While it isn’t obvious why we should we observe such a shift in the inverted condition and not the upright condition, we argue that this is at least suggestive that some features of animacy categorization, including the shift toward the “real” end of a morph continuum and the effect of orientation on animacy judgments, may be related to viewing time. As we observed in the introduction, several previous studies of how animacy is perceived have either given participants unlimited time to view images (Looser and Wheatley, 2010) or only limited presentation time to 500–1000 ms (Balas and Horski, 2012; Koldewyn et al., 2014). By including both short and long presentation times, we have observed intriguing differences in the psychometric curves relating physical position along a morph continuum spanning real and doll facial appearance to categorical judgments of face animacy. We continue in Experiment 2 by examining how these same stimulus manipulations affect animacy judgment for non-human (dog) faces.

## Experiment 2

In Experiment 2, our goal was to determine how presentation time and orientation might affect animacy categorization in non-human faces, specifically, dog faces. We hypothesized that the reduced expertise for dog faces in the general population might lead to different influences of these stimulus parameters on animacy categorization performance.

### Methods

#### Subjects

We recruited a new sample of 18 observers (nine female) to take part in this experiment. All recruitment procedures were the same as those described in Experiment 1.

#### Stimuli

**FIGURE 3:**
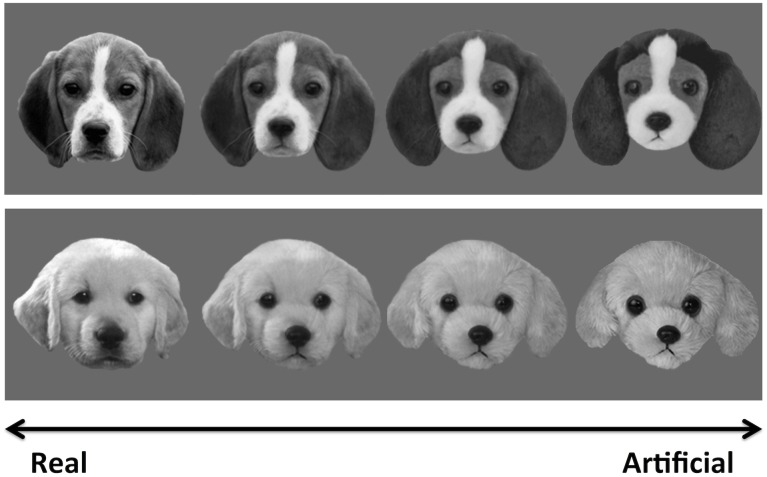
Examples of morphed real/doll dog images used in Experiment 2. As in Figure 1, these images are a subset of the full stimulus set used in this task.

As in Experiment 1, we created a set of 4 morph continua in which we blended together real faces and artificial faces. For this experiment, we used images of real dogs and plush dogs (Figure 3), cropped and formatted in the same manner as the human faces in Experiment 1. Each morph continuum depicted a unique dog breed (German shepherd, pug, e.g.) and the stimulus parameters and morphing procedures described in Experiment 1 apply to these images as well.

#### Procedure

All testing procedures were identical to those described in Experiment 1, save for the use of morphed dog images in this experiment. Otherwise, all design and display parameters, as well as response collection instructions, were the same as those described above.

### Results

**FIGURE 4:**
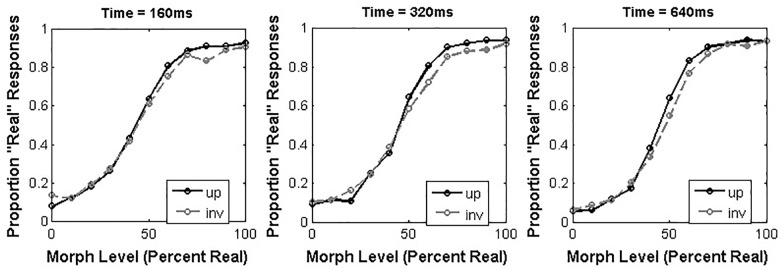
Average proportion “Real” responses to morphed real dog/doll face stimuli as a function of orientation and presentation time.

As in Experiment 1, we used curve-fitting to estimate the alpha and beta parameters that reflected the best-fit of a sigmoidal function to the proportion of “Real” responses made across morph levels in each condition (Figure 4). We present separate analyses of these parameters below.

#### Alpha Results

We analyzed the alpha values obtained from our participants across all conditions using a 3 × 2 repeated-measures ANOVA with presentation time (160, 320, and 640 ms) and orientation (upright or inverted) as within-subjects factors. This analysis revealed a significant effect of presentation time [*F*(2,34) = 3.68, *p* = 0.036, $\eta_{p}^{2}$ = 0.18], such that shorter durations led to larger alpha values. Specifically, *post hoc* tests revealed that the alpha value obtained from the 160 ms condition (*M* = 7.32, 95% CI = [3.79 – 10.86]) was significantly larger than the alpha value obtained from the 320 ms condition (*M* = 4.08, 95% CI = [0.53 – 7.62]; 95% CI of the difference between means = [1.33 – 5.16]). Neither the main effect of orientation [*F*(1,17) = 1.26, *p* = 0.28, $\eta_{p}^{2}$ = 0.069] nor the interaction between these factors [*F*(2,34) = 0.64, *p* = 0.53, $\eta_{p}^{2}$ = 0.036] reached significance.

#### Beta Results

We analyzed the beta values obtained from each participant using a 3 × 2 repeated-measures ANOVA with the same factor structure as described above for the alpha values. This analysis also revealed a main effect of presentation time [*F*(2,34) = 6.59, *p* = 0.004, $\eta_{p}^{2}$ = 0.28] such that longer presentation times led to larger beta values. Specifically, *post hoc* tests revealed that the beta value obtained at 640ms (*M* = 0.14, 95% CI = [0.084 – 0.18]) was significantly larger than the beta value obtained at 160 ms (*M* = 0.071, 95% CI = [0.065 – 0.091]; 95% CI of the difference between means = [0.002 – 0.124]). Neither the main effect of orientation [*F*(1,17) = 0.29, *p* = 0.60, $\eta_{p}^{2}$ = 0.017] nor the interaction between these factors reached significance [*F*(2,34) = 0.15, *p* = 0.086, $\eta_{p}^{2}$ = 0.26].

#### Combined Alpha and Beta Results

In order to offer a more direct comparison of the results obtained from Experiments 1 and 2, we also chose to analyze the alpha values measured in both tasks with a mixed design 3 × 2 × 2 ANOVA with presentation time and orientation as within-subject factors, and task (human faces vs. dog faces) as a between-subjects variable. While this analysis helps us avoid using a “difference of significances” to discuss the effects of task, we also wish to point out that there are some assumptions built into this analysis that may not be true. Specifically, the morph steps used to span real and artificial appearance in both tasks are ostensibly equal increments of 10%, but we have no guarantee that these actually correspond to “steps” of the same perceptual size in human and dog faces. To put it more simply, the 20% morph level for human faces may not be equivalent to the 20% morph level for dog faces, but this analysis assumes that they are. Further, the step size (which is also not guaranteed to be perceptually uniform across the continuum) may be compressed or expanded differently in the two sets of continua as well, further complicating the situation. Thus, while we think this is an important way to describe the effect of task on our results, we also urge caution in interpreting these results based on the limitations described above.

Our analysis of the combined alpha values across both experiments revealed no significant main effects of orientation [*F*(1,33) = 0.65, *p* = 0.43, $\eta_{p}^{2}$ = 0.019], presentation time [F(2,66) = 0.92, *p* = 0.40, $\eta_{p}^{2}$ = 0.14], or task [*F*(1,33) = 0.68, *p* = 0.42, $\eta_{p}^{2}$ = 0.02]. We did, however, observe significant interactions between orientation and task [*F*(1,33) = 6.34, *p* = 0.017, $\eta_{p}^{2}$ = 0.16] and presentation time and task [*F*(2,66) = 5.08, *p* = 0.009, $\eta_{p}^{2}$ = 0.13], both of which reflect the different outcomes observed in Experiments 1 and 2 as a function of task. No other interactions reached significance.

Our analysis of beta values revealed a marginally significant effect of presentation time [*F*(2,66) = 2.67, *p* = 0.076, $\eta_{p}^{2}$ = 0.075], which was qualified by a significant interaction between presentation time and task [*F*(1266) = 6.15, *p* = 0.004, $\eta_{p}^{2}$ = 0.16]. Again, this interaction reflected the different outcomes for beta values observed in Experiments 1 and 2, reported separately above. No other main effects of interactions reached significance.

## General Discussion

Across two experiments, we have found that animacy categorization is differentially affected by presentation time and orientation as a function of face species. This is consistent with results suggesting that the neural mechanisms supporting animacy judgments in human and dog faces have functional (Koldewyn et al., 2014) and neural independence (Balas and Koldewyn, 2013). The current study also extends those results by demonstrating how two simple stimulus manipulations that should affect the efficacy of face recognition in straightforward ways have different impacts on the way animacy is categorized in human and non-human faces. Specifically, there is ample evidence supporting the hypothesis that having more time to look at a face image should make you better at a wide range of recognition tasks (Shepherd et al., 1991), and likewise, inverting faces images has long been known to have profound negative consequences for many different face recognition tasks (Rossion and Gauthier, 2002). Examining how both of these constraints on efficient face processing affect the way animacy is perceived in own- and other-species faces allows us to further comment on how expertise with own-group faces leads to measurable differences in how animacy is perceived and evaluated from face images.

The simplest way to summarize the key differences we observed between Experiments 1 and 2 is to say that for human faces, orientation matters, but for non-human faces, presentation time matters. This sums up the key main effects we observed in each task and the significant interactions we observed when directly comparing the results of the two experiments to one another. This overall pattern of results is consistent with our current understanding of how expertise with a sub-group of faces (here, human faces) should impact recognition performance. When observers are looking at human faces, their extensive expertise with these images means that even very short image durations nonetheless support robust recognition, minimizing the potential to see differences in performance for faces that are visible for 160 or 640 ms. Even complex social judgments (Willis and Todorov, 2006; Borkenau et al., 2009) can be carried out at above-chance levels with far shorter image durations, so it is perhaps not surprising that giving observers about half a second longer to look at a morphed face image in our task doesn’t do very much to change the way they categorize it. By comparison, inverting human faces does seem to affect the way categorization proceeds, again, consistent with what we know about how expertise leads to specialization for face images in the upright orientation. To expand upon this point briefly, several previous studies have suggested that the strength of the inversion effect depends on face categories that the observer has expertise with, as defined by race (Hancock and Rhodes, 2008), age (Kuefner et al., 2008), and species (Parr, 2011). In each case, inversion effects are larger when observers are being presented with face images representative of their experience. Our results suggest that animacy categorization may also be affected by inversion, contrary to our previous report using only briefly presented (200 ms) images (Balas et al., 2018). As we discussed earlier, the difference between our previous report and the current study is primarily the inclusion of longer image durations in the current study, which suggests that the impact of inversion may be felt most strongly when participants have more viewing time available per image. As we emphasized above, our data are not conclusive with regard to this relationship because we are making an inference based on the difference in outcome between two studies rather than an interaction effect observed in the current report. As such, we encourage readers to take a cautious view of this interpretation of our data with regard to this point. Even so, the data from our first experiment is largely consistent with the dominant view of expert face recognition for own-group faces: fast, efficient, and subject to inversion effects.

By contrast, the results we obtained from participants’ judgments of dog faces are quite different. Critically, orientation had little effect on animacy judgments for dog faces, while presentation time did. Again, this is largely consistent with what we might expect from non-experts being asked to recognize out-group faces. First, the lack of highly efficient mechanisms for recognizing dog faces means that there is a measurable improvement between what you’re able to do in ∼160 ms and what you’re able to do in ∼640 ms. Second, inverting the image doesn’t have much of an impact because you have no expert mechanisms that have over-learned the upright orientation of dog faces. Both of these outcomes suggest that while animacy categorization can be carried out well enough with non-human face images, the manner in which it proceeds is different. There are some additional interesting features of the dog data that are worth pointing out, but as we emphasize with our human data, there are also some reasons to be cautious about making strong inferences from these features of the data. For example, we note that in contrast to human faces, the position of the PSE relative to the physical midpoint of our morph continua is consistently shifted toward the “Real” end of the spectrum. If we consider both upright (95% CI = [2.2 – 9.0]) and inverted faces (95% CI = [1.5 – 6.8]), or image durations that were short (95% CI = [3.8 – 10.9]), intermediate (95% CI = [0.5 – 7.6]) or long (95% CI = [0.4 – 6.2]), we find in each case that the psychometric curves for dog animacy appear to have the same conservative bias toward more restrictive “Real” judgments that is characteristic of human face animacy ratings in many studies. Why should this be, and why should shorter image durations exacerbate this shift when we have argued above that for human faces it might do the exact opposite? We cannot make a strong statement about this point, but it is an intriguing issue to consider for future work. We also note that the marginal effect of task on beta values is somewhat intriguing in that it appears to favor a steeper psychometric curve for dog animacy judgments, which is somewhat at odds with what we might expect. The steepness of the curve is usually associated with the reliability of sensory performance (Strasburger, 2001) and so we might expect that observers would have more consistency for human faces than dog faces. Again, we cannot make a strong statement about this effect given that it is at best a weaker trend, but this suggests that there may be important issues to consider when we examine how animacy is perceived in different categories. For example, the different material properties (e.g., fur vs. skin) that distinguish real faces from artificial faces in each species category may mean that there are measurable differences in the perceptual increments that comprise each continuum between real and artificial faces across different face categories, or that observers apply different response criteria to animacy judgments based on the visibility of specific small-scale features. The raw image similarity between different steps of a putatively uniform continuum spanning variation in high-level appearance attributes like animacy has yet to be carefully evaluated or controlled, and this is also likely a critically important issue for future work to consider.

What do these results imply about the theoretical issues we discussed regarding the contribution of top–down and bottom–up processes to animacy perception and the specificity of animacy perception as considered within the domain of face recognition. The simplest conclusion we can draw is that our results demonstrate that face animacy perception is not unitary – different stimulus manipulations impact performance differently as a function of species. We argue that this has important implications for the relationship between animacy perception and social evaluation. Specifically, though dogs are certainly social animals that humans interact with frequently, our results suggest that human face animacy perception is governed by mechanisms that do not apply to dog faces. Critically, the lack of an impact of stimulus duration on human face animacy perception speaks to the potential for rapid bottom-up processes to help execute animacy categorization in service of subsequent mind perception processes that may modulate a range of social judgments. We suggest that this means that while the animacy of human faces (and possibly bodies) can modulate social processes like attentional orienting, it may be the case that the animacy of other-species faces does not change performance in such tasks. That is, observers’ capacity for mind perception may be fine-tuned to rapidly extract relevant animacy information for human faces, but less able to do the same work for non-human faces. This makes animacy perception similar to other “thin-slice” judgments in that robust and reliable estimates of social variables can be made from highly constrained stimuli, but our data also suggest limits to that ability to may be defined by experience and social relevance.

Our data also has implications for how observers decide to interact with agents that may be real or artificial. The resilience of the step-like boundary between real and artificial human faces to image duration in particular suggests that observers uncertainty regarding the animate status of a face likely does not change much, meaning that feelings of uncanny-ness or creepiness probably do not fluctuate during initial contact with a new face. Instead, our results suggest that initial animacy categorization is consistent for human faces after short or long intervals. Thus, a range of important decisions regarding social interaction, including approach-avoid judgments (Oosterhof and Todorov, 2008), imitative behaviors (Nehaniv and Dautenhahn, 2007), and social evaluation (Balas and Tupa, 2018), likely follow a similar stable trajectory in microgenetic terms (Sekuler and Palmer, 1992). Another way to put this is to say that our data suggest that face-specific mechanisms work quickly and accurately to categorize face animacy so that animacy can in turn inform decisions about what to expect from a social agent, how to behave during an interaction with it, and how best to model the agent’s mind to support accurate inferences regarding it’s cognitive states. Admittedly it is challenging to build a bridge between low-level psychophysical results like ours and these higher-level aspects of social cognition and interactive behavior, but as we mentioned in the introduction, the relationship between low-level sensory processes and top–down influences on visual behavior remains an important issue in animacy perception research. Our results primarily contribute to the goal of understanding the first steps of evaluating animacy in face images, but nonetheless reveal the tools the visual system has available to it so that the perceived animacy of agents can be used to guide a wide range of behaviors and judgments.

These complexities notwithstanding, our results highlight some important features of how animacy is perceived in human faces. Critically, by examining non-human animacy judgments with the same paradigm, we are able to make some clearer statements about the specialization of mechanisms applied to human faces in service of animacy judgments. Our results provide an important bridge between several disparate examinations of how animacy is evaluated and categorized in human and non-human faces images, supporting the hypothesis that human animacy judgments rely on expert mechanisms for face processing.

## Ethics Statement

This study was carried out in accordance with the recommendations of the North Dakota State University IRB with written informed consent from all subjects. All subjects gave written informed consent in accordance with the Declaration of Helsinki. The protocol was approved by the North Dakota State University IRB.

## Author Contributions

BB designed the experiments, analyzed the data, and wrote the manuscript. AA collected and analyzed the data and edited the manuscript.

## Conflict of Interest Statement

The authors declare that the research was conducted in the absence of any commercial or financial relationships that could be construed as a potential conflict of interest. The reviewer FC and handling Editor declared their shared affiliation.
